# Cardiac catheterization addressing early post-operative complications in congenital heart surgery—a single-center experience

**DOI:** 10.1186/s43044-020-00117-6

**Published:** 2020-11-23

**Authors:** Saud Bahaidarah, Jameel Al-Ata, Gaser Abdelmohsen, Naif Alkhushi, Mohamed Abdelsalam, Mohammed Mujahed, Osman Al-Radi, Ahmed Elassal, Zaher Zaher, Ahmad Azhar, Ahmed M. Dohain

**Affiliations:** 1 Pediatric Cardiology Division, Department of Pediatrics, King Abdulaziz University, P.O Box 80215, Jeddah, 21589 Saudi Arabia; 2 Pediatric Cardiology Division, Department of Pediatrics, Cairo University, Cairo, 11562 Egypt; 3 Cardiac Surgery Division, Department of Surgery, King Abdulaziz University, Jeddah, Kingdom of Saudi Arabia; 4 Cardiology Department, Benha University, Benha, Egypt; 5 Cardiothoracic Surgery Department, Zagazig University, Zagazig, Egypt

## Abstract

**Background:**

Cardiac catheterization after congenital heart surgery may play an important role in the diagnosis and management of patients with a complicated or unusual post-operative course. The main objective of this study was to evaluate the safety, efficacy, and outcome of cardiac catheterization performed in the early post-operative period following congenital heart surgery. All patients who underwent cardiac catheterization after congenital heart surgery during the same admission of cardiac surgery from November 2015 to May 2018 were included in the study.

**Results:**

Thirty procedures were performed for 27 patients (20 interventional and 10 diagnostic). The median age of the patients was 15 months (15 days to 20 years), median weight was 8.2 kg (3.4 to 53 kg), and median time from surgery was 3 days (0–32 days). Eleven procedures were performed for 11 patients on extracorporeal membrane oxygenation (ECMO) support. The main indications for catheterization included the inability to wean from ECMO (10 procedures) and cyanosis (10 procedures). Interventional procedures included angioplasty using stents (10 procedures, success rate of 90%), angioplasty using only balloons (2 procedures, success rate of 50%), and occlusion for residual shunts (8 procedures, success rate of 100%). No mortality was recorded during any procedure. Vasoactive–inotropic score had significantly decreased 48 h after catheterization when compared to pre-catheterization scores (*p* = 0.0001). Moreover, 72% of patients connected to ECMO support were successfully weaned from ECMO after catheterization. Procedural complications were recorded in 3 interventional procedures. Survival to hospital discharge was 55.5% and overall survival was 52%. Patients on ECMO support had a higher mortality than other patients.

**Conclusion:**

Cardiac catheterization can be performed safely in the early post-operative period, and it could improve the outcome of the patient (depending on the complexity of the cardiac lesions involved).

## Background

Cardiac catheterization is one of the most important tools used in the management of congenital heart diseases. There is minimal risk of adverse events in most cardiac catheterization procedures in the current era [1–5]. However, cardiac catheterization may carry a relatively significant risk of major adverse events, in certain conditions particularly in critically ill patients after congenital heart surgery. Despite this risk, the role of catheterization is considered vital in critically ill patients with a residual lesion or unexpected progress, post-cardiac surgery. In some instances, identification of the cause of clinical deterioration after congenital heart surgery could not be feasible by non-invasive modalities, like echocardiography. The use of echocardiography is limited by either the poor acoustic windows or the delayed sternal closure that is frequently encountered in the early post-operative period [6]. In these patients, cardiac catheterization could be helpful for hemodynamic evaluation and identification of significant residual lesions. As residual lesions after congenital heart surgery might carry a high risk for poor outcome, cardiac catheterization could provide a detailed evaluation of residual lesions and helps ensure that a proper decision was made for either further intervention during the same catheterization procedure or referral for redo surgery [7–9]. Data and experience in this field are growing [10–13].

The aim of this study was to evaluate the safety, efficacy, and outcome of cardiac catheterization performed in the post-operative period (mostly on an emergency basis) and during the same admission after surgery.

## Methods

This is a retrospective cohort study that included all patients who underwent cardiac catheterization (diagnostic or interventional) during the same admission after congenital heart surgery in the period from November 2015 to May 2018. The study was approved by our institutional ethical committee. Written consents from the patients’ legal guardians were already taken on admission for potential participation in future research. The collected data included age, weight, diagnosis, type of surgery, indication of cardiac catheterization, type of cardiac catheterization, time from surgery, the need for redo surgery, the type of redo surgery, duration of mechanical ventilation, inotropic score (IS), vasoactive–inotropic score (VIS), duration of ECMO support, duration of hospital and pediatric intensive care unit (PICU) stay, procedure complications, and outcomes. IS was calculated as dopamine dose (μg/kg/min) + dobutamine dose (μg/kg/min) + 100 × epinephrine dose (μg/kg/min). VIS was calculated as IS + 10 × milrinone dose (μg/kg/min) + 10,000 × vasopressin dose (Units/kg/min) + 100 × norepinephrine dose (μg/kg/min) [14].

The success of the interventional procedures was also reported and defined as follows: For angioplasty (ballooning or stenting), procedure success was defined as an increase in the vessel diameter to more than 75% of the adjacent normal vessel or at least 50% from the pre-procedural diameter. For occlusion, procedure success was defined as the absence of significant residual shunting as seen by angiography [11, 15]. The impact of catheterization procedures on patients’ clinical conditions like duration of mechanical ventilation, IS, PICU length of stay, and successful weaning from ECMO support was also recorded.

## Statistical analysis

Statistical analysis was performed using SPSS version 26 software (IBM SPSS Statistics for Windows, version 26.0. Armonk, NY: IBM Corp). Data were expressed as median and range (minimum-maximum) for numerical data and number and percentage for categorical data. Comparisons between groups were calculated using nonparametric Mann–Whitney and Wilcoxon signed rank tests for numerical data and *χ*^2^ tests for categorical data. Survival analysis was done using Kaplan–Meier log-rank univariate analysis.

## Results

### Patients’ characteristics

Twenty-seven patients underwent 30 cardiac catheterization procedures (10 diagnostic and 20 interventional). The median age at the time of cardiac catheterization was 15 months (ranging from 15 days to 20 years). The median time from cardiac surgery was 3 days ranging from 0 days (same day of surgery) to 32 days. Out of 30 procedures, six procedures were performed for six patients immediately after surgery (3 interventional and 3 diagnostic), 12 more procedures were performed in the 1st week post-surgery (5 diagnostics, 7 interventions), and 2 procedures were performed in the 2nd week post-surgery. The median weight at the time of cardiac catheterization was 8.2 kg (3.4–53 kg). Fourteen procedures were performed for 12 patients with single ventricle physiology and 10 procedures in nine patients with conotruncal anomalies. Two genetic syndromes were identified in this cohort, the first one being DiGeorge syndrome and the other Williams syndrome. Twenty-three procedures were performed for 21 patients on mechanical ventilation and inotropic support; the median duration of mechanical ventilation was 12 days (2–89 days). Eleven procedures were done for 11 patients on ECMO support, and the median duration for ECMO was 5 days, range 2–7 days. There was no significant difference between patients that underwent diagnostic cardiac catheterization and those that underwent interventional procedure regarding age, weight, diagnosis, time from surgery, mechanical ventilation, VIS, or ECMO support. Patients’ characteristics are illustrated in Table 1.

**Table 1:** Patients’ characteristics

	Number = 27
**Age (months)**	15 (0.49–241)
**Weight (kg)**	8.2 (3.4–53)
**Diagnosis,** ***n*** **(%)**	
Single ventricle	12 (44.4)
Conotruncal anomaly: TOF, DORV, PA-VSD, IAA	9 (33.3)
Others: TAPVC, ALCAPA, Shone’s complex	6 (22.2)
**Syndromes,** ***n*** **(%)**	2 (7.4)
Williams	1 (3.7)
DiGeorge	1 (3.7)
**Cardiac catheterization,** ***n*** **(%)**	
Diagnostic	9 (33.3)
Intervention	18 (66.7)
**Time from surgery to catheterization, days**	3 (0–32)
**Redo surgery after catheterization,** ***n*** **(%)**	7 (25.9)
VSD fenestrations	3 (11.1)
Fontan fenestration	1 (3.7)
Baffle augmentation	2 (7.4)
Arch repair	1 (3.7)
**ECMO,** ***n*** **(%)**	11 (40.7)
**ECMO duration, days**	5 (2–7)
**Mechanical ventilation,** ***n*** **(%)**	21 (77.8)
**Total duration of mechanical ventilation, days**	12 (2–89)
**Inotropic support,** ***n*** **(%)**	21 (77.8)
**Vasoactive–inotropic score at the time of catheterization**	25 (5–90)
**PICU admission,** ***n*** **(%)**	16 (59.2)
**PICU stay, days**	16 (1–99)
**Hospital stay, days**	20 (2–330)

*Abbreviations: kg* kilograms, *TOF* tetralogy of Fallot, *DORV* double outlet right ventricle, *PA-VSD* pulmonary atresia with ventricular septal defect, *IAA* interrupted aortic arch, *TAPVC* total anomalous pulmonary venous connection, *ALCAPA* anomalous left coronary artery from the pulmonary artery, *ECMO* extracorporeal membrane oxygenation, *PICU* pediatric intensive care unit

### Indication for cardiac catheterization

Inability to wean from ECMO/cardiopulmonary bypass (CPB) and cyanosis were the most frequent indications (Table 2).

**Table 2:** Indication of cardiac catheterization

Indications of cardiac catheterization, ***n*** (%)	All procedures (***n*** = 30)
Inability to wean ECMO/CPB	10 (33.33)
Cyanosis	10 (33.33)
Low cardiac output	2 (6.66)
RV failure/increased peritoneal catheter or pleural drainage	5 (16.66)
Heart failure symptoms	3 (10)

*Abbreviations: ECMO* extracorporeal membrane oxygenation, *CPB* cardiopulmonary bypass, *RV* right ventricle

### Catheterization procedures

For the intervention group, angioplasty through stenting was performed in 10 procedures, six of them were for pulmonary artery branches. The success rate in the stenting group was 90% (stent embolization into the LPA was reported in one patient that required surgical retrieval).

Angioplasty through ballooning was performed in two procedures: one for a kink in a modified Blalock–Thomas–Taussig shunt (BTT) that was successful with improvement of oxygen saturation after the procedure. The other was for stenosis of a pulmonary venous confluence to left atrial connection after repair of total anomalous pulmonary venous connection (TAPVC) that failed to decrease the pressure gradient or heart failure symptoms, requiring a redo surgery. In all angioplasty procedures, the median balloon size to stenosis diameter ratio was 2.5 (1.8–3).

**Fig. 1:**
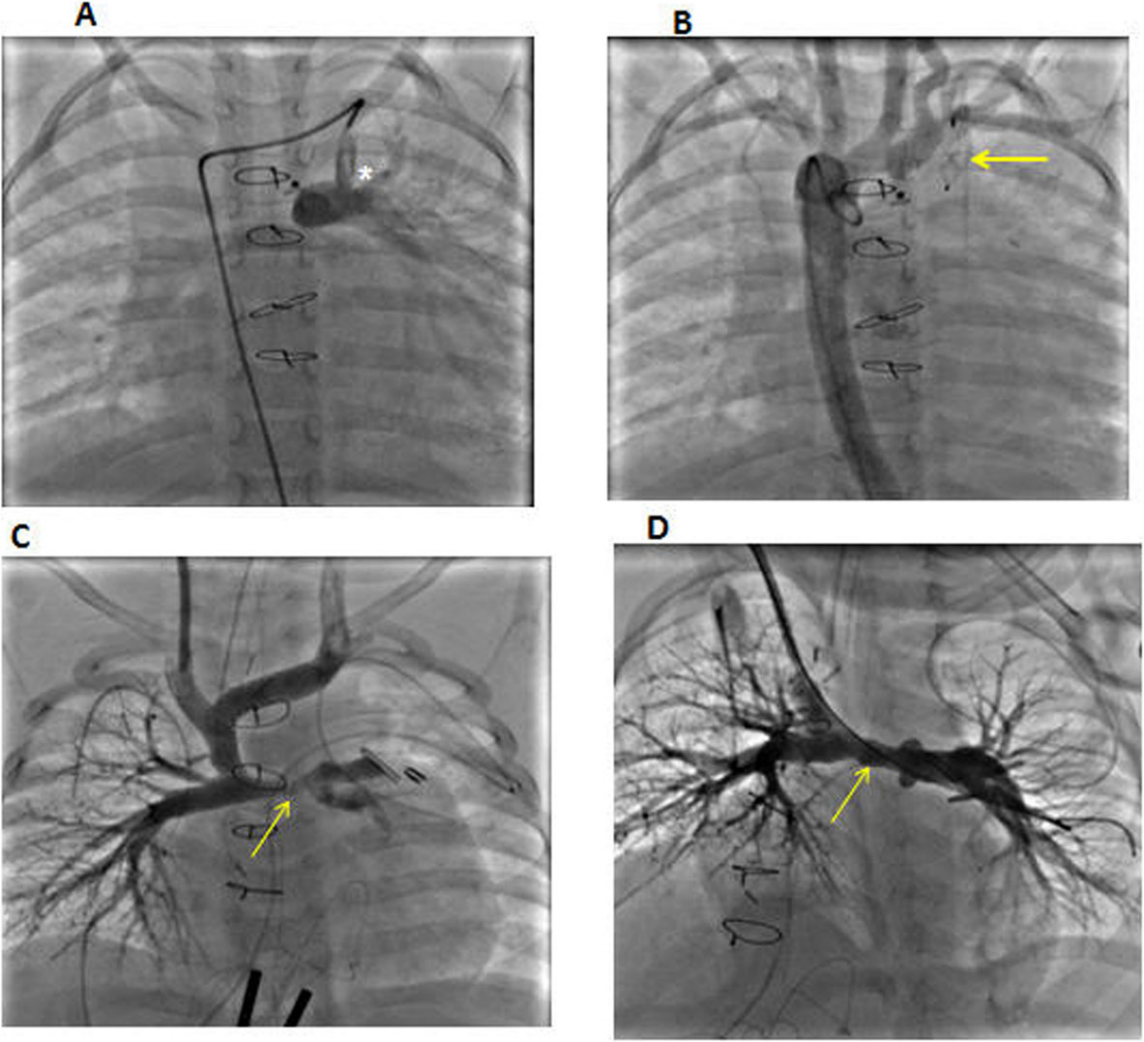
**a** Selective angiography for residual MBT shunt (white asterisk) in a patient after Fallot tetralogy repair; the patient developed heart failure symptoms due to significant left to right shunt. **b** The same patient after occlusion of the MBT shunt with vascular plug (yellow arrow) with no residual flow seen after aortic angiography. **c** Significant LPA stenosis (yellow arrow) with decreased left lung vascularity in another patient after Glenn surgery; the patient developed significant desaturation (68–73%) after surgery. **d** The same patient after LPA stenting (yellow arrow) with the improvement of left lung vascularity and patient saturation increased to 85%. MBT shunt, modified Blalock–Taussig shunt; LPA, left pulmonary artery

**Fig. 2:**
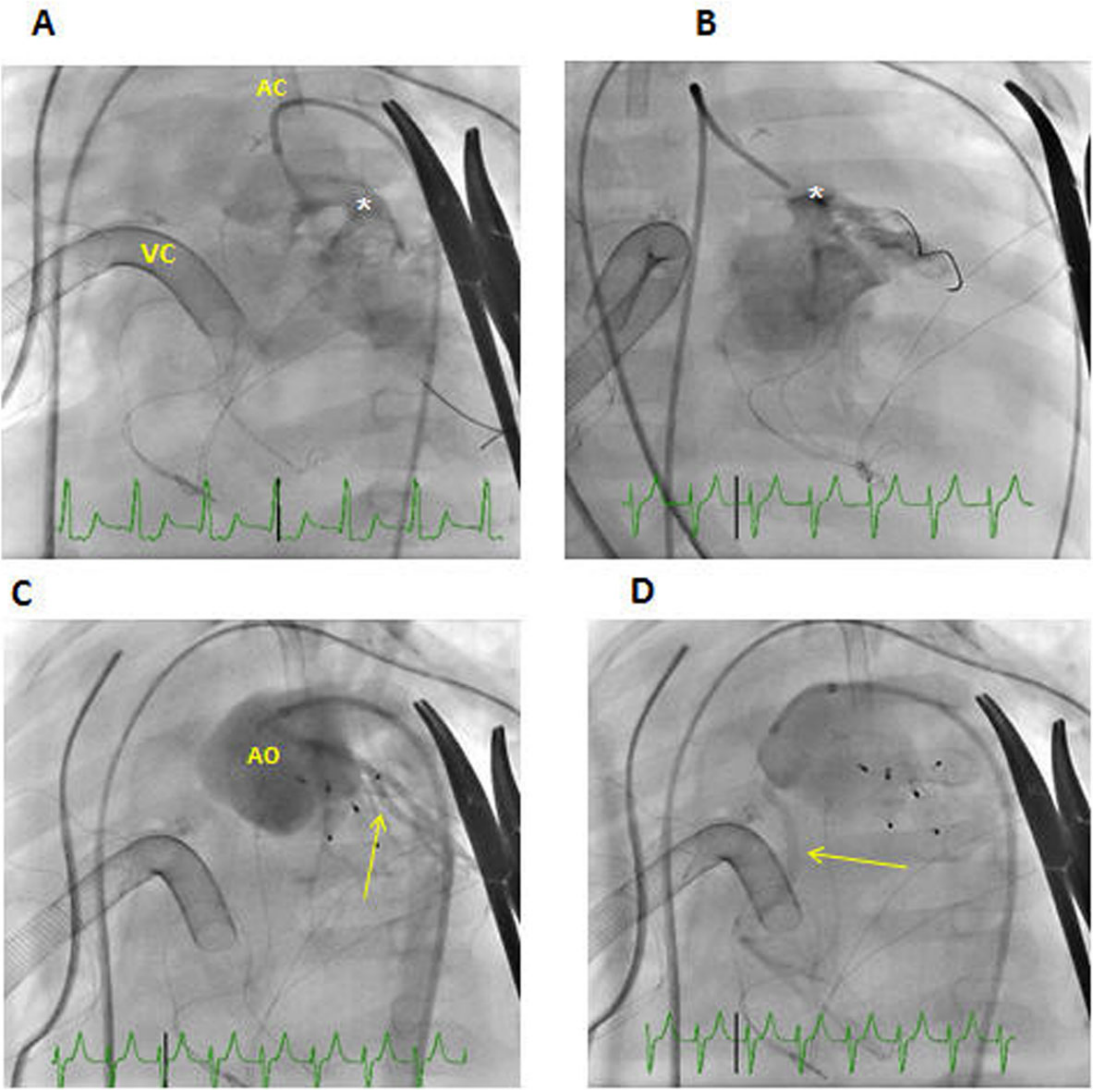
Cardiac catheterization performed for a 6-month-old baby that presented late with DORV, subpulmonic VSD, intramural left coronary artery, and hypoplastic aortic arch after arterial switch operation on ECMO support. The patient developed an iatrogenic AO to RV fistula that led to severe coronary steal, severe cardiac dysfunction, and inability to wean from ECMO support. **a** Contrast injection in the neo-aorta revealed a large AO-RV fistula (white asterisk) with a coronary steal (coronary arteries are not seen with neo-aortic injection). **b** Selective injection of the AO-RV fistula (white asterisk).**c**, **d** Neo-aortic injection after fistula is occluded with vascular plugs showed no residual fistula and no more coronary steal as coronary arteries are seen well after neo-aortic injection (yellow arrows). Three days later, the patient was successfully disconnected from ECMO, and contractility progressively improved and the patient was discharged home. DORV double outlet right ventricle, VSD ventricular septal defect, VC venous cannula, AC arterial cannula, AO aorta, RV right ventricle, ECMO extracorporeal membrane oxygenation

Occlusion was performed in 8 procedures with a 100% success rate. Interventional procedures and complications related to cardiac catheterizations are illustrated in Table 3. Examples of interventional cardiac catheterization procedures are illustrated in Figs. 1 and 2. Redo surgery was performed for 3 patients after interventional cardiac catheterization. In this cohort, diagnostic cardiac catheterization was performed for 10 patients, four of which were connected to ECMO support. Based on information obtained from cardiac catheterization, four patients (40%) underwent redo surgery (1 arch repair/connected to ECMO, 1 Fontan fenestration, 1 VSD closure/connected to ECMO, and 1 redo for enlargement of confluence to left atrial anastomosis after repair for total anomalous pulmonary venous connection).

**Table 3:** Success and complications of cardiac catheterization

Interventional procedures and their success rate (***n*** = 20)		
Type of procedure	***n*** (%)	Success, ***n*** (%)
***Angioplasty/stenting***	10 (50%)	9 (90%)
Pulmonary arteries stenting	6	
RVOT stenting	2	
RCA stenting	1	
Pulmonary venous baffle stenting	1	
***Angioplasty/ballooning***	2 (10%)	1 (50%)
Pulmonary venous baffle	1	
Modified BT shunt	1	
***Closure***	8 (40%)	8 (100%)
MAPCA	2	
Fontan fenestration	2	
Pulmonary AVMS	1	
Modified BT shunt	1	
AO-RVOT fistula	1	
Antegrade pulmonary flow in Fontan	1	
**Complications of cardiac catheterization in all procedures (*n*** **= 30)**		
Cardiac arrest during procedure, *n* (%)		1 (3.33)
Embolization of stent/device, *n* (%)		1 (3.33)
Arterial thrombosis, *n* (%)		1 (3.33)

*Abbreviations: RVOT* right ventricular outflow tract, *RCA* right coronary artery, *BT shunt* Blalock–Taussig shunt, *MAPCA* major aorto-pulmonary collateral arteries, *AVMS* arterio-venous malformations, *AO* aorta

### Outcome

In this cohort, 21 patients were mechanically ventilated and on inotropic support. The VIS significantly decreased 48 h after cardiac catheterization (*p* = 0.0001, Table 4). The duration of mechanical ventilation and PICU stay were unsurprisingly longer after cardiac catheterization because most of the procedures were done so early in the first few days after surgery while patients were connected to mechanical ventilation and admitted in PICU (8 of the interventional procedures were performed within 24 h of the surgery).

**Table 4:** Outcome of cardiac catheterization

	Before catheterization	After catheterization	***p*** value
**Vasoactive–inotropic score**	25 (5–90)	10 (0–90)	0.001
**Duration of mechanical ventilation**	1 (0–17)	10 (0–80)	0.003
**PICU length of stay**	1 (0–57)	13 (0–78)	0.001
**Survival**			
Survival to hospital discharge, *n* (%)	15 (55.5)		
Overall survival, *n* (%)	14 (52%)		
Successful weaning off ECMO, *n* (%)	8 (72.7)		
Survival of ECMO patients, *n* (%)	4 (36.3)		

*Abbreviations*: *PICU* pediatric intensive care unit, *ECMO* extracorporeal membrane oxygenation

Out of the total 30 procedures, there was no complication reported during the transfer of patients from the operating room or PICU to the cardiac catheterization laboratory. Three interventional procedures had complications during cardiac catheterization, namely, a cardiac arrest that required cardiopulmonary resuscitation, a stent embolization, and a post-procedural arterial thrombosis of the femoral artery that improved on heparin infusion. There was no reported mortality during any of the procedures.

The median duration of hospital stay was 20 days (2–330 days) and for PICU stay was 16 days (1–99 days). There was no significant difference between the diagnostic group and interventional group regarding PICU stay, hospital stay, or the need for redo surgery. Regarding survival, the overall survival till the time of the study was 52% (14 out of 27 patients). Survival to discharge was 55.5% (15 out of 27 patients, one patient died 1 year after the procedure from infective endocarditis). Table 4 illustrates the outcome of cardiac catheterization among this cohort.

### Procedures performed on ECMO support

**Fig. 3:**
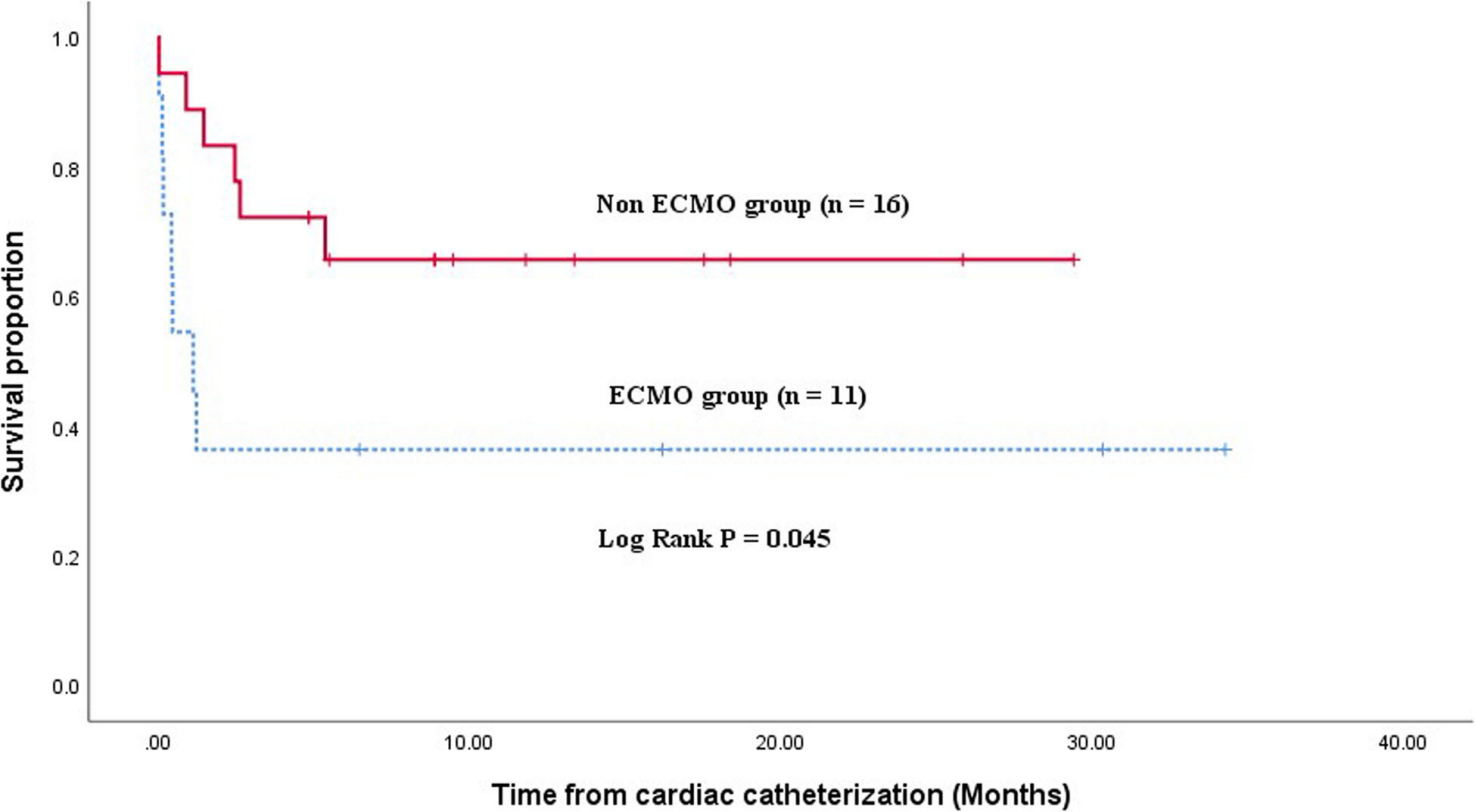
Kaplan–Meier survival curve demonstrating less survival among patients that underwent cardiac catheterization on ECMO support. ECMO extracorporeal membrane oxygenation

Eleven procedures were performed for 11 patients on ECMO support (4 diagnostic and 7 interventional). Eight patients (72.7%) were successfully disconnected from ECMO support. Five of them (45.4%) survived to hospital discharge (one out of those five patients died a year after the procedure with infective endocarditis). Kaplan–Meier univariate analysis demonstrated that patients who underwent cardiac catheterization on ECMO support had a lesser survival rate than others (Fig. 3).

## Discussion

In the early post-operative period after congenital heart surgery, some children become critically ill either due to residual lesions, severe myocardial dysfunction, low cardiac output, missed (underestimated or masked) lesions, or iatrogenic complications. This can lead to inability to wean from CPB/ECMO support. Cardiac catheterization may play a role not only in the diagnosis but also in the management of such patients [10, 16]. It was believed that at least 6 weeks should pass post-cardiac surgery before any attempt for interventional cardiac catheterization to allow for scar tissue formation around the suture lines and anastomotic sites despite no studies to support this rationale [11]. In this cohort, we aimed to study the safety, success, and outcome of cardiac catheterization performed after congenital heart surgery and during the same admission (all procedures were done < 5 weeks post-cardiac surgery). In this cohort, a multidisciplinary team including the surgeon, intensivist, cardiac anesthesiologist, cardiac interventionist, ECMO team, and respiratory therapist was ready to intervene at any time during catheterization or patients’ transfer to the catheterization laboratory. No complications were reported during patients’ transfer to the catheterization laboratory, and this was concordant with previously published data [11]*.*

### Diagnostic cardiac catheterization

Diagnostic cardiac catheterization in the early post-operative period can play an important role in the evaluation of the adequacy of surgical repair and detection of significant residual lesions that may affect the hemodynamics and post-operative course. Hence, catheterization can help in proper decision-making regarding subsequent management plans either conservative treatment, redo surgery, or further catheter intervention. In this study, diagnostic cardiac catheterization was able to detect residual cardiac lesions of hemodynamic significance in 40% of patients, and based on the data collected during catheterization, the decision for redo surgery was taken. Importantly, interventional cardiac catheterization resulted in a significant reduction of the required inotropic support. We also noticed no significant difference between patients that underwent diagnostic catheterization and those that underwent interventional procedures with regard to the hospital stay, PICU stay, complications, and survival, Nicholson et al. reported similar results in their study [10].

### Angioplasty and vascular stenting

Theoretically speaking, angioplasty (ballooning/stenting) in the early post-operative period may cause catastrophic disruption of the freshly formed sutures, due to the long-standing belief of having to allow time for scar formation around vascular suture lines. In this cohort, angioplasty was performed in 12 procedures, 9 of them done on suture lines with no reported vascular tear or suture disruption and this was concordant with a report by Zahn et al. [11] who attributed that the distensibility of Prolene filaments can afford elongation up to 34% before disruption and enlargement of the circumference of the suture line (increased distance between parallel throws) in response to balloon inflation. Zahn et al. concluded that continuous Prolene suture lines can be expanded safely without disruption when using balloon to stenosis ratio ≤ 2.5/1 [11]. In this cohort, the median balloon size to stenosis diameter ratio was 2.5 for angioplasty procedures. Rosales et al. reported a 20% mortality due to vascular disruption after balloon angioplasty of the branch pulmonary arteries in the early post-operative period [15]. In a majority of cases, stent placement is better than balloon dilatation because it prevents the recoil giving better results for a considerable period of time [11].

### Cardiac catheterization for the patient on ECMO support

Several reports studied the safety of catheter intervention for patients on ECMO support [7, 17–19]. When compared to our study, the results are less promising in our cohort. Although we were able to successfully disconnect 72% of patients from ECMO support, survival to discharge was 45.4% and overall survival was only 36% in this high-risk group. This is likely the result of their complex anatomy, associated co-morbidities, or unsuccessful surgical intervention. It is not clear if the early cardiac catheterization contributed to successful ECMO discontinuation or if it was the natural progress of the patients. This needs to be explored separately and with a larger sample. Similar results were reported by Kojima et al. (overall survival rate was 29%), while Desjardins et al. reported a lower survival rate (overall survival was 14%). Many studies reported higher overall survival rates (43%, 56%, 64%, and 69%) [7, 16, 17, 19, 20].

## Limitations of the study

This study is only presenting a small number of patients with different disease severities for short period. It is also limited by its retrospective design and lack of control group comparison.

## Conclusion

Cardiac catheterization after congenital heart surgery is a safe and effective procedure that may play an important role in the diagnosis and management of patients with complicated or unexplained post-operative course, and can improve the outcome that is mostly dependent on the complexity of cardiac lesions and associated co-morbidities. Further studies on a larger scale are recommended.

## Data Availability

All data were available at King Abdulaziz University Hospital.

## Abbreviations

AC: Arterial cannula
ALCAPA: Anomalous left coronary artery from the pulmonary artery
AO: Aorta
AVMS: Arterio-venous malformations
BTT shunt: Blalock–Thomas–Taussig shunt
CPB: Cardiopulmonary bypass
DORV: Double outlet right ventricle
ECMO: Extracorporeal membrane oxygenation
IAA: Interrupted aortic arch
IS: Inotropic score
LPA: Left pulmonary artery
MAPCAS: Major aorto-pulmonary collateral arteries
MBT shunt: Modified Blalock–Taussig shunt
PA: Pulmonary atresia
PICU: Pediatric intensive care unit
RCA: Right coronary artery
RV: Right ventricle
RVOT: Right ventricular outflow tract
TAPVC: Total anomalous pulmonary venous connection
VC: Venous cannula
VIS: Vasoactive–inotropic score
VSD: Ventricular septal defect

## References

[1] Moore JW, Vincent RN, Beekman RH (2014). Procedural results and safety of common interventional procedures in congenital heart disease: initial report from the national cardiovascular data registry. J Am Coll Cardiol..

[2] Feltes TF, Bacha E, Beekman RH (2011). Indications for cardiac catheterization and intervention in pediatric cardiac disease: a scientific statement from the American Heart Association. Circulation..

[3] Dancea A, Justino H, Martucci G (2013). Catheter intervention for congenital heart disease at risk of circulatory failure. Can J Cardiol..

[4] Lin CH, Hegde S, Marshall AC (2014). Incidence and management of life-threatening adverse events during cardiac catheterization for congenital heart disease. Pediatr Cardiol..

[5] Kugler JD, Beekmani RH, Rosenthal GL (2009). Development of a pediatric cardiology quality improvement collaborative: from inception to implementation. From the Joint Council on Congenital Heart Disease Quality Improvement Task Force. Congenit Heart Dis..

[6] Elassal AA, Eldib OS, Dohain AM, Abdelmohsen GA, Abdella AH, Al-Radi OO (2019). Delayed sternal closure in congenital heart surgery: a risk-benefit analysis. Heart Surg Forum..

[7] DesJardins SE, Crowley DC, Beekman RH, Lloyd TR (1999). Utility of cardiac catheterization in pediatric cardiac patients on ECMO. Catheter Cardiovasc Interv..

[8] Abraham BP, Gilliam E, Kim DW, Wolf MJ, Vincent RN, Petit CJ (2016). Early catheterization after initiation of extracorporeal membrane oxygenation support in children is associated with improved survival. Catheter Cardiovasc Interv..

[9] Howard TS, Kalish BT, Wigmore D (2016). Association of extracorporeal membrane oxygenation support adequacy and residual lesions with outcomes in neonates supported after cardiac surgery∗. Pediatr Crit Care Med..

[10] Nicholson GT, Kim DW, Vincent RN, Kogon BE, Miller BE, Petit CJ (2014). Cardiac catheterization in the early post-operative period after congenital cardiac surgery. JACC Cardiovasc Interv..

[11] Zahn EM, Dobrolet NC, Nykanen DG, Ojito J, Hannan RL, Burke RP (2004). Interventional catheterization performed in the early postoperative period after congenital heart surgery in children. J Am Coll Cardiol..

[12] Elassal AA, Al-Radi OO, Dohain AM, Abdelmohsen GA, Al-Ebrahim KE, Eldib OS (2020). Excess nonhemorrhagic pleural drainage after surgery for congenital heart diseases: single center experience. J Card Surg..

[13] Ismail MF, Elmahrouk AF, Arafat AA (2020). Bovine jugular vein valved xenograft for extracardiac total cavo-pulmonary connection: the risk of thrombosis and the potential liver protection effect. J Card Surg..

[14] Gaies MG, Gurney JG, Yen AH (2010). Vasoactive–inotropic score as a predictor of morbidity and mortality in infants after cardiopulmonary bypass*. Pediatr Crit Care Med..

[15] Rosales AM, Lock JE, Perry SB, Geggel RL (2002). Interventional catheterization management of perioperative peripheral pulmonary stenosis: balloon angioplasty or endovascular stenting. Catheter Cardiovasc Interv..

[16] Kojima T, Imamura T, Osada Y et al (2018) Efficacy of catheter interventions in the early and very early postoperative period after CHD operation. Cardiol Young.:1–5. 10.1017/S1047951118001452 10.1017/S1047951118001452 PMC6316360 30175700

[17] Booth KL, Roth SJ, Perry SB, Del Nido PJ, Wessel DL, Laussen PC (2002). Cardiac catheterization of patients supported by extracorporeal membrane oxygenation. J Am Coll Cardiol..

[18] Dohain AM, Abdelmohsen G, Elassal AA, Elmahrouk AF, Al-Radi OO (2019). Factors affecting the outcome of extracorporeal membrane oxygenation following paediatric cardiac surgery. Cardiol Young..

[19] Callahan R, Trucco SM, Wearden PD, Beerman LB, Arora G, Kreutzer J (2015). Outcomes of pediatric patients undergoing cardiac catheterization while on extracorporeal membrane oxygenation. Pediatr Cardiol..

[20] Panda BR, Alphonso N, Govindasamy M, Anderson B, Stocker C, Karl TR (2014). Cardiac catheter procedures during extracorporeal life support: a risk–benefit analysis. World J Pediatr Congenit Hear Surg..

